# phMRI: methodological considerations for mitigating potential confounding factors

**DOI:** 10.3389/fnins.2015.00167

**Published:** 2015-05-07

**Authors:** Julius H. Bourke, Matthew B. Wall

**Affiliations:** ^1^Centre for Psychiatry, The London School of Medicine and Dentistry, Wolfson Barts Institute for Preventive Medicine, Queen Mary University of LondonLondon, UK; ^2^Imanova Centre for Imaging Sciences, Imperial College London, Hammersmith HospitalLondon, UK; ^3^Division of Brain Sciences, Imperial College LondonLondon, UK

**Keywords:** pharmocological MRI, pharmaco fMRI, phMRI, methodology, neuroscience, cognitive neuroscience, psychopharmacology, phfMRI

## Abstract

Pharmacological Magnetic Resonance Imaging (phMRI) is a variant of conventional MRI that adds pharmacological manipulations in order to study the effects of drugs, or uses pharmacological probes to investigate basic or applied (e.g., clinical) neuroscience questions. Issues that may confound the interpretation of results from various types of phMRI studies are briefly discussed, and a set of methodological strategies that can mitigate these problems are described. These include strategies that can be employed at every stage of investigation, from study design to interpretation of resulting data, and additional techniques suited for use with clinical populations are also featured. Pharmacological MRI is a challenging area of research that has both significant advantages and formidable difficulties, however with due consideration and use of these strategies many of the key obstacles can be overcome.

## Introduction

Twenty-five years after its conception, functional MRI (fMRI) remains a stalwart technique in cognitive neuroscience research. This well-established method of investigating “activation” of the brain can also be combined with pharmacological agents; a technique (possibly contentiously; Sauter and Rudin, 2000) referred to as pharmacological MRI or pharmaco-fMRI (phMRI). This technique is of increasing interest in drug discovery (Wise and Tracey, 2006) and in the exploration of neurological and mental disorder (Honey and Bullmore, 2004). The use of MRI to study drug effects shares many challenges with standard fMRI (including issues of reliability and reproducibility; Zuo and Xing, 2014; Zuo et al., 2014) but also presents other unique difficulties. The purpose of this brief review is to outline some of the main considerations that need to be addressed when designing and carrying out phMRI studies.

**Figure 1:**
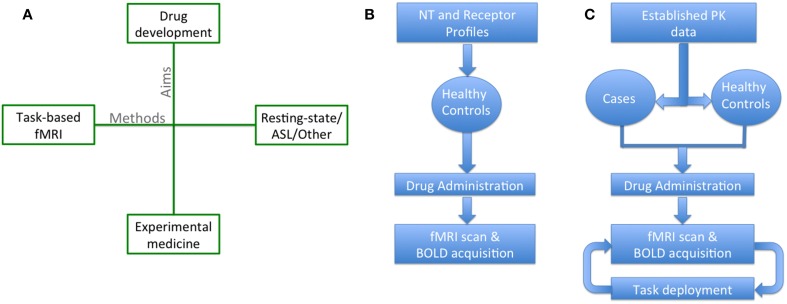
**Schematics representing different kinds of phMRI studies. (A)** The aims of phMRI (vertical axis) can be divided into “challenge” studies aimed more at drug development (often using novel compounds, and occurring in early-phase clinical trials, using healthy volunteers), and “activation” studies that can be classed as experimental medicine (more often using an established/marketed compound, and may use clinical samples). Similarly the methods (horizontal axis) can be divided into those focused on “standard” task-based fMRI, and those using non-task methods (resting-state fMRI, ASL, or other MRI contrasts). **(B)** A schematic of a typical “challenge” study focusing on drug development. **(C)** A schematic of a typical “activation” or experimental medicine study. NT, Neurotransmitter; fMRI, functional Magnetic Resonance Imaging; BOLD, Blood Oxygen Level Dependent; ASL, Arterial Spin Labeling.

Broadly, phMRI studies can be classified on two dimensions (Figure 1A): the general aims of the study, and the methods employed. In terms of the investigational aims, “challenge” studies (Figure 1B) phMRI represents a potentially powerful tool in drug discovery, providing real time neurophysiological data on drug action (Upadhyay et al., 2011) as part of early-phase clinical trials, and more often uses healthy participants. The primary aim here is to investigate the mechanism of drug action. In contrast, “activation” studies (Figure 1C) phMRI studies fall under the general classification of experimental medicine. They are typically more hypothesis driven (Iannetti and Wise, 2007; Carhart-Harris et al., 2014) and use established drugs, with known mechanisms of action, in order to investigate neural systems and probe individual symptoms and disorders, sometimes using clinical populations. In terms of the methods used, a broad distinction can be drawn between those studies that use standard task-based fMRI, and others that use non-task methods such as resting-state fMRI, Arterial Spin Labelling (ASL), Magnetic Resonance Spectroscopy (MRS) or other methods. The precise applicability of the issues and methodological techniques outlined below to any particular study depends somewhat on the quadrant that it occupies in Figure 1A (though some may straddle multiple quadrants, for example it is relatively common practice to include task and resting scans in a single session). Previous reviews (e.g., Iannetti and Wise, 2007; Jenkins, 2012) have largely focused on drug discovery studies; however important additional factors apply when conducting different kinds of studies, particularly when using clinical samples.

## Limitations in the interpretation of bold response

The most frequently used technique in fMRI studies involves the measurement of changes in the Blood Oxygen Level Dependent (BOLD) signal. BOLD is a naturally occurring contrast representing local changes in the ratio of oxygenated and deoxygenated hemoglobin (Buxton et al., 1998; Friston et al., 2000). The BOLD response to a given stimulus is therefore a proxy measure of neural activity and relies on a cascade of cellular events; a relationship known as “neurovascular coupling” (Logothetis et al., 2001). The use of BOLD as a measure of underlying neural activity assumes that this cascade and its relationship with the cerebral vasculature are intact. The BOLD response is therefore dependent on baseline activity (from which changes can be measured), intracellular pathways allowing communication with the vasculature, and vascular responsivity.

Introducing a pharmacological agent to BOLD studies can potentially disrupt this neurovascular coupling and in so doing, render results more difficult to interpret (Iannetti and Wise, 2007). Drugs may disrupt local neuronal firing, the cellular processes giving rise to the BOLD response, or modify baseline neural or vascular activity across the entire brain, potentially resulting in over/underestimations of effects (Hyder et al., 2002). These considerations are particularly acute when using phMRI in the development of novel agents, where drug effects on neurovascular coupling are typically unknown, or when using BOLD signal to infer activity at neurotransmitter receptors (Breiter et al., 1997).

**Figure 2:**
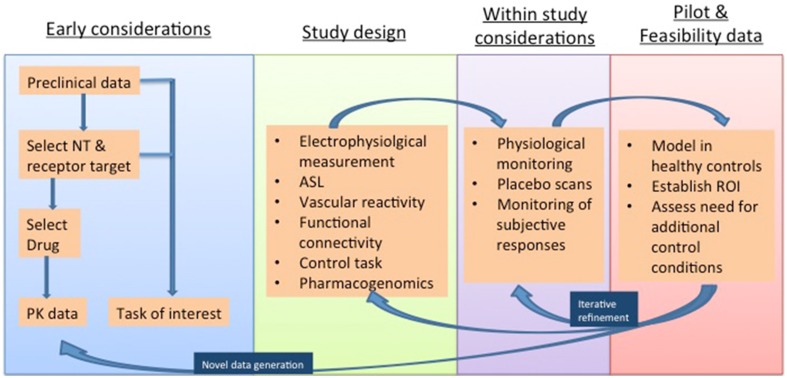
**Methodological considerations in phMRI studies illustrated in a proposed timeline and considering both “challenge” studies of novel agents and “activation” studies of established agents deployed for the investigation of task paradigms and disease states**. NT, Neurotransmiter; PK, pharmacokinetic; ASL, Arterial Spin Labelling; ROI, Region Of Interest.

Many neurotransmitters are active in the systemic vasculature and local neurovasculature, either directly, or by way of modulating downstream signaling pathways (Jenkins, 2012). It is therefore vital to take these factors into account in study design. The remainder of this review highlights strategies that can mitigate these confounding factors, and thereby bolster the interpretation of phMRI investigations. Such “control” conditions are discussed below and summarized in Figure 2.

## Choice of target neurotransmitter and experimental drug

The involvement of different neurotransmitters in systemic processes varies. An early phMRI study of cocaine (Breiter et al., 1997) highlighted the importance of vascular considerations in the context of dopamine, which is vasoactive and plays an important role in the control of systemic blood pressure (Amenta et al., 2000; Tayebati et al., 2011). Dopamine has two families of receptors; D1-like, and D2-like. Arterioles and capillaries vasodilate upon activation of D1-like receptors, while capillary activation of D2-like receptors results in vasoconstriction (Choi et al., 2006). Alterations in BOLD signal may therefore reflect positive haemodynamic changes coupled to agonism of D1-like receptors or negative effects coupled to D2-like receptors (Chen et al., 2005, 2010; Dixon et al., 2005; Choi et al., 2006, 2010; Shih et al., 2009).

Another instructive example is caffeine, which effects alertness and performance (e.g., Wesensten et al., 2002; Brunye et al., 2012) but is also a powerful cerebral vasoconstrictor (Mathew and Wilson, 1985). The former results from antagonism of adenosine A_1_ and A_2A_ receptors, while the latter effect is due to selective activity at A_2A_ receptors (Diukova et al., 2012). Since the relative density of these two receptor subtypes varies across the brain, caffeine can have variable effects in different brain regions. Some authors have shown that caffeine boosts the BOLD response (Mulderink et al., 2002) while others show consistent reductions (Diukova et al., 2012). Even for such a relatively selective and widely-studied drug as caffeine, the situation is complex.

For non-selective drugs, in brain regions where there is a preponderance of one receptor subtype there is likely to be a relative excess of vasodilation/constriction. Potentially, in regions where the two subtypes are equally abundant, no net change might be observed.

Few drugs are selective for one receptor subtype or indeed one neurotransmitter. With a single pharmacological agent it is therefore possible to observe positive, negative, or no effects on BOLD that may actually reflect more global vascular effects, rather than task-related changes. Early consideration in phMRI study design may help overcome these issues but more often than not, additional control strategies are required to account for these possibilities. These can be most effectively designed with adequate physiological and pharmacokinetic data on the neurotransmitter and drug of choice.

## Matching drug effect to regional bold signal change

Target neurotransmitters and receptor profiles are typically understood at an early stage of drug development and this can be used in combination with knowledge of brain receptor distribution. Data on absorption rates and time to maximum serum concentrations is available for clinical drugs, and is vital in ascertaining the timing of administration. Additional data should be sought on time to peak drug effect, onset, and duration of action that will assist in establishing whether serial and chronic administration is required, which may be used in a challenge phMRI study, or single and one-off dosing, which may be preferred in activation studies (Bloom et al., 1999). For chronic administration, associating drug dose with the effect on the BOLD response can further validate this approach. While knowledge of the pharmacokinetics of drugs employed in phMRI is a significant advantage, this is often unavailable for novel agents in challenge phMRI studies and in these instances, phMRI may itself be vital in obtaining this data.

### Patient group heterogeneity

Research in patient groups requires additional considerations to control for confounding factors that may weaken the generalizability of the conclusions. Additional drugs may be co-prescribed, necessitating multiple exclusion criteria and leading to slow study recruitment. Although scientifically desirable, it is not always possible, or indeed ethical, to ensure that patient groups are pharmacologically “pure.” In these instances it is vital that co-prescribed medication is not in danger of independently affecting the BOLD response, the task of interest, or of interacting with the drug of interest. Variations in the illness state may further affect the data acquired and illness severity may also vary between subjects. These issues can be partly overcome through the use of an additional “healthy” control group, enabling modeling of drug effects and the identification of regions of interest (ROI), if not already known, in a more homogeneous and tightly-controlled group. Data from healthy subjects can be important, but may not fully generalize to patient populations, as the autoregulation of neurophysiology is such that chronic responses to sustained alterations in neurotransmitters and their receptors are potentially different to those in health.

### Monitoring and correcting for physiological parameters

Monitoring of physiological parameters in phMRI studies is necessary in order to rule out systemic effects that may drive the observed task-specific signal changes. This is a simple addition to most studies, as physiological parameters (e.g., heart rate, oxygen saturation) are routinely used for monitoring during scanning. However, these data can also serve as an independent source of noise that can be removed during analysis, and specific methodology has been developed for this purpose (e.g., Glover et al., 2000).

### Using separate placebo scans

Inferences about task-related BOLD signal changes are benefitted greatly by comparison with signal during rest periods, but this comparison remains under the experimental (drug) condition. The use of a placebo scan, with the same paradigm deployed in each session, may serve to strengthen inferences. Even where single oral administration or intravenous infusion is employed, the use of two scans (separated by days or weeks) is preferable to a single scan session employing both placebo and active drug (McKie et al., 2005; Deakin et al., 2008). This is because the placebo would need to be administered first (in order to ensure that observed effects do not reflect ongoing drug effects during placebo administration), which introduces an order effect. Separate scanning sessions are also able to control for other variables (e.g., scanner thermal drift) that may potentially confound the measurement of drug-related effects. In the case of challenge phMRI studies, especially those involving patient groups, this may be problematic, as it is possible that physiological changes (e.g., disease progression) have taken place between scans. This is also the case where chronic administration of a drug is required, as the potential for significant between-session variability in parameters that relate to the participant and the scanning environment will also be harder to control for. Nevertheless, the use of more than one scanning session introduces the possibility of within and between group/participant comparisons, which adds statistical power and helps to additionally control for MRI-related factors affecting the BOLD response.

### Regions of interest and control tasks

The use of a ROI approach (examining networks that have already been established as responsive to a particular task or sensory stimulus) can strengthen study design and in the context of activation studies this approach is often feasible and preferable. Although this increases the power to detect regional drug effects, other effects detectable with a whole brain approach may be missed. However, a ROI approach allows for the introduction of an additional task, unrelated to the paradigm of interest, and capable of activating unrelated networks. For example, a visual task might be added to an activation study examining affective changes (Murphy et al., 2009). The absence of any observable effect on BOLD response to the visual task between the placebo and drug scans suggests that drug effects on the main task of interest cannot be accounted for by global vascular (or other) effects. The power of this strategy to rule out extraneous factors as an explanation for observed changes increases with the number of control tasks employed (Pinel et al., 2007) but still cannot completely rule out non-specific regional effects produced by the primary task (Iannetti and Wise, 2007). A low threshold should be set for the detection of drug effects on the control task in order to avoid the underestimation of differences (Iannetti and Wise, 2007).

### Subjective responses

For drugs that involve measurable subjective effects, (e.g., drowsiness, alertness, other psychoactive effects), recording these at predetermined time points during acquisition of scanning data allows for the calculation of correlations between known subjective effects of a drug and regional brain activation (Breiter et al., 1997; Murphy and Mackay, 2011). Previous studies have expanded on this concept using a regression approach to model the time course of drug effects in the brain (Anderson et al., 2002). Although elegant, this method assumes that these subjective effects are entirely, or mostly, neuronal in origin. It is also a method limited to drugs with known immediate psychological effects, which may limit this approach to agents with more rapid action.

### Electrophysiological recordings

A more optimal but perhaps less practical method of measuring task and drug-related changes in neuronal activity is through the use of electrophysiological measures, such as EEG and MEG. These provide a direct measurement of neural activation with excellent temporal resolution. EEG has poor spatial resolution due to skull conductivity, whilst MEG has similar temporal resolution to EEG but is better able to distinguish between sources of activation (Murphy and Mackay, 2011). These measures add additional data on task and drug-related activity, recording different aspects of neuronal activity to the BOLD signal. It is possible (though technically challenging) to record BOLD and EEG data simultaneously; though MEG requires a separate session. The decision to use EEG/MEG as an additional source of “control” data may therefore be more dependent on the nature of the stimulus or task employed than the presence of the experimental drug condition (Iannetti and Wise, 2007).

## Vascular controls

The ability to control for and measure vascular differences between drug and placebo conditions is desirable in phMRI studies in order to compensate for the limitations of the interpretation of BOLD response to a drug challenge. These methods also render additional drug and disease-related data that may be independently desirable.

### Assessing baseline perfusion

Arterial spin labeling (ASL) is a means of labeling arterial blood magnetically, resulting in an additional naturally occurring contrast medium (Williams et al., 1992). ASL can be used to acquire estimations of baseline and dynamic changes in CBF. This method involves a less favorable signal to noise ratio, covers less brain volume and has worse temporal resolution than BOLD measurements (Aguirre et al., 2002). However, it is more sensitive to slower changes in CBF that are seen in response to drug administration and may better localize neuronal activity changes (Pfeuffer et al., 2002), as well as providing more directly quantitative data than BOLD fMRI. Its advantage in phMRI is in being able to assess changes in baseline perfusion that may confound task-specific changes in BOLD signal (Cohen et al., 2002; Bendlin et al., 2007). ASL data is increasingly thought of as essential to phMRI study design (Iannetti and Wise, 2007; Murphy and Mackay, 2011).

### Assessing the rate of cerebral O2 metabolism and vascular reactivity

BOLD signal is a product of changes in CBF, CBV and the local rate of O_2_ metabolism (CMRO_2_). The latter is an event that occurs upstream of CBF and CBV responses and so provides better spatial resolution in terms of assessing where changes in neuronal activity occur than the composite measure of BOLD signal alone (Bandettini and Wong, 1997). This can be achieved experimentally by comparing BOLD response before and during an experimentally-induced rise in CBF, achieved through the inspiration of carbon dioxide (CO_2_) (Davis et al., 1998; Hoge et al., 1999). The higher partial pressure of CO_2_ results in robust cerebral vasodilation and increased perfusion without increases in CMRO_2_ and so BOLD signal. In studies employing drug and placebo scans, this paradigm can be deployed once during both sessions (van der Zande et al., 2005; Pattinson et al., 2007).

## Gene polymorphism

Differences in the metabolism and degradation of neurotransmitters may have a bearing on the activity of drugs and drug effects. Enzymatic degradation of the monoamines is a case in point; the activity of catecholamine-O-methyltransferase (COMT) is one enzyme involved in this process and is subject to genetic polymorphisms. Variations in the val(158)met gene produce variations in COMT activity and so variations in neurotransmitter activity (Mattay et al., 2003). Such polymorphisms could influence drug effects on task-specific activity but may also produce variations in the drug effect on components of the BOLD response. An additional “control” condition may therefore be employed through the analysis of known polymorphisms for a given neurotransmitter of interest. COMT val(158)met has been used in this regard in both phMRI and positron emission tomography (PET) imaging (Mattay et al., 2003; Zubieta et al., 2003). The use of gene analysis also provides the potential for stratification of patient groups so that more specific pharmacological targets can be identified. phMRI therefore has applications in the growing field of pharmacogenomics (Hariri and Weinberger, 2003; Wise and Tracey, 2006; Bifone and Gozzi, 2011).

## Functional connectivity

One particular advantage of non-task based fMRI (i.e., resting-state fMRI) is it enables analysis of functional connectivity (Murphy and Mackay, 2011). The benefit here is that estimates of functional connectivity rely more upon the degree to which activity in different brain areas correlate, rather than the simple amplitude of activity in any one region. As such, connectivity measures may be less vulnerable to global drug effects than BOLD signal changes used in conventional analyses. There are a great number of methodological approaches to the analysis of resting-state fMRI data (Cole et al., 2010) and many have been shown to be sensitive markers of disease states and drug effects. However, the precise functional nature of resting-state brain networks is unresolved and this combined with the data-driven nature of many analysis strategies impedes robust interpretations.

## Conclusion/summary

phMRI continues to be a novel, informative, and important method in drug discovery and basic/clinical neuroscience. It is not without its shortfalls, but with appropriate considerations in methodology and study design it has the potential to fill the gap between MRI and PET methodologies on the frontier of neuroscientific and pharmacological research.

### Conflict of interest statement

Matthew B. Wall is employed by Imanova Ltd., a private company that performs contract research work for the pharmaceutical industry, as well as supporting academic research. The authors declare that the research was conducted in the absence of any commercial or financial relationships that could be construed as a potential conflict of interest.
